# Protein kinases modulate store-operated channels in pulmonary artery smooth muscle cells

**DOI:** 10.1186/1423-0127-18-2

**Published:** 2011-01-06

**Authors:** I-Shan Chen, Zen-Kong Dai, Donald G Welsh, Ing-Jun Chen, Bin-Nan Wu

**Affiliations:** 1 Department of Pharmacology, School of Medicine, College of Medicine, Kaohsiung Medical University, Kaohsiung, Taiwan; 2 Department of Pediatrics, School of Medicine, College of Medicine, Kaohsiung Medical University, Division of Pediatric Pulmonology and Cardiology, Kaohsiung Medical University Hospital, Kaohsiung, Taiwan; 3 Smooth Muscle Research Group and Department of Physiology and Pharmacology, University of Calgary, Calgary, Alberta, Canada

## Abstract

**Background:**

This study investigates whether protein kinase G (PKG), protein kinase A (PKA) and protein kinase C (PKC) are involved in the regulatory mechanisms of store-operated channel (SOC) in pulmonary arteries.

**Methods:**

Pulmonary artery smooth muscle cells (PASMCs) were enzymatically dissociated from rat intralobar pulmonary arteries. Whole cell, cell-attached and inside-out patch-clamp electrophysiology were used to monitor SOCs in isolated PASMCs.

**Results:**

Initially the Ca^2+^-ATPase inhibitor cyclopiazonic acid (CPA, 10 μM) initiated a whole cell current that was reduced by the SOC blocker SKF-96365 (10 μM). Subsequent work using both cell-attached and whole cell configurations revealed that the PKG and PKA inhibitors, KT5823 (3 μM) and H-89 (10 μM), also stimulated SOC activity; this augmentation was attenuated by the SOC blockers SKF-96365 (10 μM) and Ni^2+ ^(0.1 mM). Finally using the inside-out configuration, the PKC activator phorbol 12-myristate 13-acetate (PMA, 10 μM) was confirmed to modestly stimulate SOC activity although this augmentation appeared to be more substantial following the application of 10 μM inositol 1,4,5-triphosphate (Ins(1,4,5)P_3_).

**Conclusions:**

SOC activity in PASMCs was stimulated by the inhibition of PKG and PKA and the activation of PKC. Our findings suggest that the SOC could be a substrate of these protein kinases, which therefore would regulate the intracellular concentration of calcium and pulmonary arteriopathy via SOC.

## Background

Intracellular calcium ([Ca^2+^]_i_) is an important second messenger responsible for many physiological functions including contraction, cell growth and gene expression. Many agonists increase [Ca^2+^]_i _by mobilizing intracellular Ca^2+ ^stores, such as the sarcoplasmic (SR) or endoplasmic (ER) reticulum. To maintain Ca^2+ ^signaling, these intracellular Ca^2+ ^stores must be refilled and as such many agonists are thought to activate specialized plasma membrane channels termed store-operated channels (SOCs) [1]. By definition, SOCs are Ca^2+^-permeable cation channels which are activated by depletion of intracellular Ca^2+ ^stores [2]. The activation of SOCs is often termed 'capacitative Ca^2+ ^entry (CCE)', as the principal function of these Ca^2+ ^channels is to refill the internal stores, as if they were in essence capacitors [3]. Inhibitors of the sarco-endoplasmic reticulum Ca^2+^-ATPase pump (SERCA) are the most common tools used to deplete the intracellular Ca^2+ ^stores and consequently activate these unique channels.

It is generally accepted that SOCs play an important role in regulating smooth muscle contraction and cellular proliferation in the resistance vasculature [4,5]. In a similar, although less documented manner, SOCs have also been coupled to the genesis of pulmonary vascular tone and pulmonary artery smooth muscle cell (PASMC) proliferation [6]. Given their functional importance and their role in severe pulmonary arteriopathies, there is considerable interest in defining how SOCs are regulated in PASMCs [7]. To date, literature specific to PASMCs has prominently stressed a role for IP_3_, PIP_2 _and other lipid products in the activation of these channels. While important, few pulmonary studies have ventured beyond these confines to electrically address other aspects of SOC regulation, including the role of protein kinase G (PKG), protein kinase A (PKA) and protein C (PKC). This is surprising given the rich nature of the research performed on smooth muscle cells derived from resistance arteries isolated from the coronary, mesenteric and hepatic circulation [4,8,9].

The main objectives of this study were to isolate and characterize a SOC current in pulmonary artery myocytes and determine whether protein kinases (i.e. PKG, PKA and PKC) are involved in the activation of SOCs in PASMCs. Whole cell, cell-attached and inside out patch-clamp electrophysiology were used to monitor SOCs and the effects of various agents known to modulate protein kinases were recorded. Like smooth muscle cells isolated from the coronary, mesenteric and hepatic circulation, SOCs in PASMCs were stimulated by PKG and PKA inhibition and PKC activation. The functional significance of these findings is discussed.

## Materials and methods

### Animal procedures and tissue preparations

All procedures and protocols were approved by the Animal Care and Use Committee at Kaohsiung Medical University. Briefly, female Sprague-Dawley rats (250-350 g) were sacrificed with an overdose of urethane (1.25 g/kg) via intraperitoneal injection. Lungs were carefully removed and placed in cold phosphate-buffered saline containing (in mM): 122 NaCl, 1 MgCl_2_, 0.5 KH_2_PO_4_, 10 HEPES, 5 KCl, 0.5 NaH_2_PO_4_, 11 Glucose, 0.1 EGTA, 0.1 CaCl_2_, with pH adjusted to 7.4 with NaOH. Intralobar resistance pulmonary arteries (internal diameter 300-400 μm) were dissected free of the surrounding tissue and cut into 1 mm segments.

### Preparation of isolated pulmonary artery myocytes

Pulmonary artery smooth muscle cells (PASMCs) from rat intralobar pulmonary arteries were enzymatically isolated as follows. Arterial segments were placed in a warm (37°C) cell isolation medium containing (in mM) 122 NaCl, 1 MgCl_2_, 0.5 KH_2_PO_4_, 10 HEPES, 5 KCl, 0.5 NaH_2_PO_4_, 11 Glucose, 0.1 EGTA (pH 7.4, NaOH) for 20 min. After this equilibration step, arterial segments were initially incubated (37°C) in 1 mg ml^-1 ^papain and 0.85 mg ml^-1 ^dithioerythritol for 20-25 min. After enzyme treatment, the tissue was washed three times in ice-cold isolation medium and triturated with a fire-polished pipette to release the myocytes. Cells were stored in ice-cold isolation medium for use on the same day.

### Patch-clamp electrophysiology

SOC currents in PASMCs were recorded in voltage-clamp mode using whole cell, cell-attached and inside-out configurations [10]. When employing whole cell patch-clamp electrophysiology, PASMCs were placed in a recording dish and perfused with a bath solution containing (in mM): 120 sodium methanesulfonate, 20 Ca(OH)_2_, 0.5 3,4-diaminopyridine, 10 HEPES and 10 glucose (pH 7.4, HCl). A recording electrode pulled from borosilicate glass (resistance, 4-7 MΩ for whole cell recordings; 8-12 MΩ for cell-attached and inside-out patches) was coated with sticky wax to reduce capacitance [11,12] and backfilled with pipette solution containing (in mM): 138 CsOH, 2.5 EGTA, 1 Ca(OH)_2 _(free internal [Ca^2+^] ~100 nM as calculated using EQCAL software), 10 HEPES and 2 Na_2_ATP (pH 7.2, HCl). This pipette was gently lowered onto a PASMC, negative pressure was briefly applied to rupture the membrane and a gigaohm seal was obtained. Cells were voltage clamped at 0 mV while resting membrane currents were recorded on an Axopatch 700A amplifier (Axon Instruments, Union City, CA, USA). Cells were subsequently equilibrated for 25 min and then exposed to a series of voltage ramps (-100 mV to +100 mV, 0.2 Vs^-1^) or step protocols (20 mV increments from -80 to +20 mV for 200 ms). These voltage protocols were performed under resting conditions and in the presence of cyclopiazonic acid (CPA, 10 μM) ± 1-[β-(3-(4-Methoxyphenyl)propoxy)-4-methoxyphenethyl]-1H-imidazole HCl (SKF-96365, 10 μM) [13,14], (9S,10R,12R)-2,3,9,10,11,12-hexahydro-10-methoxy-2,9-dimethyl-1-oxo-9,12-epoxy-1H-diindolo-[1,2,3-fg:30,20,10-kl]pyrrolo[3,4-i][1,6]benzodiazocine-10-carboxylic acid methylester (KT5823, 3 μM) or N-[2-((p-Bromocinnamyl)amino)ethyl]-5-isoquinolinesulfonamide (H-89, 10 μM). Whole cell currents were then filtered at 1 kHz (low-pass Bessel filter), digitized at 5 kHz and stored on a computer for subsequent analysis with Clampfit 9.0. A 1 M NaCl-agar salt bridge between the bath and the Ag-AgCl reference electrode was used to minimize offset potentials [12,15]. All electrical recordings were performed at room temperature.

When using the inside-out and the cell-attached patch clamp configurations to monitor single channel activity, recording pipettes were backfilled with a solution containing (in mM): 126 CsCl, 10 HEPES, 11 Glucose, 1.5 CaCl_2_, 10 TEA, 5 4-AP, 0.0002 Iberiotoxin, 0.1 DIDS, 0.1 Niflumic acid and 0.005 Nifedipine (pH 7.2, NaOH). The bath solution for the inside-out configuration contained (in mM): 18 CsCl, 108 C_2_H_3_CsO_2_, 1.2 MgCl_2_, 10 HEPES, 11 Glucose, 1 BAPTA, 0.48 CaCl_2_, 1 Na_2_ATP and 0.2 NaGTP (pH 7.2, Tris). The bath solution for the cell-attached configuration contained (in mM): 126 KCl, 1.5 CaCl_2_, 10 HEPES, 11 Glucose and 0.01 Nifedipine (pH 7.2, Tris). Following a 25 min equilibration period, single channel activity in excised patches was recorded at -80 mV, filtered at 100 Hz and digitized at 50 kHz. These recordings were collected under resting conditions and in the presence of phorbol 12-myristate 13-acetate (PMA, 10 μM), inositol-1,4,5-triphosphate (Ins(1,4,5)P_3_, 10 μM), KT5823 (3 μM) and H-89 (10 μM).

### Data analysis and statistics

Whole cell SOC currents were analyzed from the baseline-to-peak amplitude within 50 ms. Single SOC current amplitudes were calculated from idealized traces of at least 60 s in duration using the 50% threshold method and analyzed using Clampfit 9.0 as previously described [8]. For single channel analysis, SOC activity (NP_o_) was determined from continuous gap-free data using Clampfit 9.0. The NP_o _was calculated from the following equation: NP_o _= (*Σt*_*i*_*i*)/T, where *i *is the number of channels open, *t*_*i *_is the open time for each level *i *and T is the total time of analysis. Data are expressed as means ± SE, *n *indicating the number of cells. Repeated measures analysis of variance (ANOVA) compared values at a given voltage. When appropriate, a Tukey-Kramer pairwise comparison was used for *post hoc *analysis. ANOVA followed by Dunnett's test was performed to statistically compare the open probability of SOCs. *P *≤ 0.05 was considered statistically significant.

### Chemicals

Buffer reagents, papain, dithioerythritol, H-89, KT5823, PMA, CPA, Ins(1,4,5)P_3, _NiCl_2 _and SKF-96365 were obtained from Sigma-Aldrich Chemical Co. (St Louis, MO, USA). All drugs and reagents were dissolved in distilled water unless otherwise noted. CPA, PMA and KT5823 were dissolved in dimethylsulphoxide at 10 mM. Serial dilutions were made in phosphate-buffered solution to a final solvent concentration of ≤0.01%.

## Results

### CPA evoked whole cell currents in rat PASMCs

**Figure 1:**
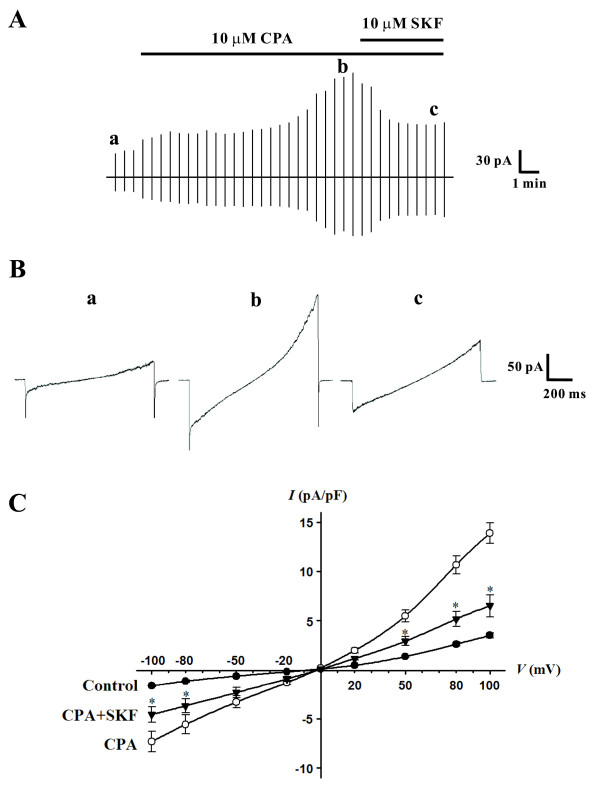
**Cyclopiazonic acid (CPA) evokes an SOC conductance**. Using whole cell patch clamp electrophysiology, PASMCs were voltage clamped and then periodically exposed to voltage ramps (-100 mV to +100 mV) in the absence and presence of 10 μM CPA ± 10 μM SKF-96365 (SKF). **A**, original traces showing that CPA activates whole cell SOC currents in PASMCs. SKF-96365 was added to the perfusate 12 min after CPA. **B**, individual *I-V *relationships under resting conditions (**a**, Control), and in the presence of CPA (**b**) and SKF-96365 (**c**). **C**, mean *I-V *relationships of whole cell SOC currents. Data are means ± SE, n = 6. * denotes significant difference CPA alone.

Our investigation of SOCs in PASMCs first began by monitoring the whole cell currents evoked by CPA. This assessment involved the application of voltage ramps (-100 mV to +100 mV, 0.2 Vs^-1^) every 30 s from a holding potential of 0 mV in order to inactivate voltage-dependent Na^+ ^and Ca^2+ ^channels. The recording solutions dictate that inward currents at negative potentials should be the result of Ca^2+ ^and Na^+ ^influx, while outward current at positive potentials should be putatively generated by Cs^+ ^efflux [13]. Figure 1 illustrates that bath application of 10 μM CPA induces a whole cell SOC current that displays modest outward rectification and which reverses at -2 ± 1 mV (n = 6). In the presence of CPA, current density at -100 mV and +100 mV peaked at -7.3 ± 1.1 pA pF^-1 ^and 13.9 ± 1.1 pA pF^-1 ^(n = 6), respectively. These values were significantly greater (P < 0.01) than control (-1.6 ± 0.1 pA pF^-1 ^and 3.5 ± 0.3 pA pF^-1 ^at -100 mV and +100 mV, respectively). CPA evoked whole cell currents were subsequently attenuated by the addition of SKF-96365 (10 μM, a SOC inhibitor) to the perfusate. In the presence of CPA and SKF-96365, current density at -100 mV and +100 mV was 4.6 ± 0.8 pA pF^-1 ^and 6.5 ± 1.1 pA pF^-1^, respectively.

### Activation of SOCs by a PKG inhibitor

**Figure 2:**
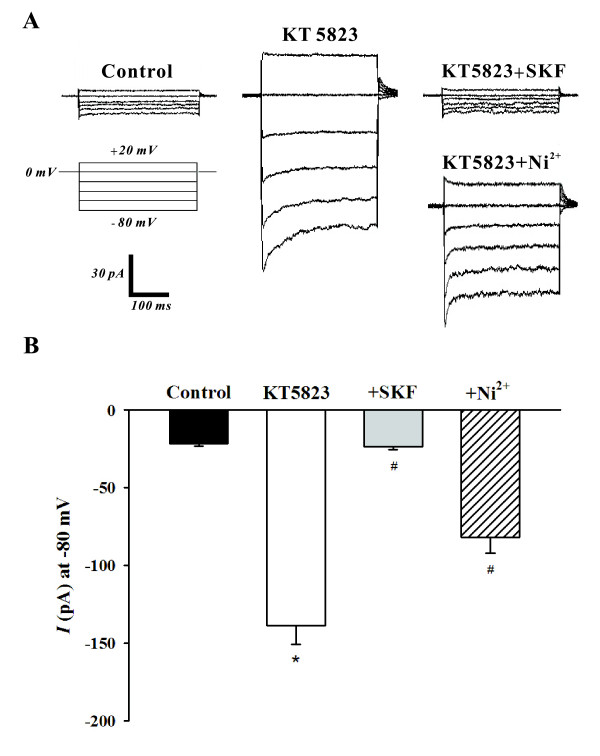
**PKG inhibition by KT5823 augments SOC whole cell currents**. Using whole cell patch clamp electrophysiology, PASMCs were voltage clamped and then periodically exposed to a step protocol (-80 mV to +20 mV, 20 mV increments, 300 ms duration) in the absence and presence of 3 μM KT5823 ± 10 μM SKF-96365 (SKF) or 0.1 mM Ni^2+^. A representative trace and summary data can be found in **A **&**B**, respectively. Data are means ± SE, n = 6. * and # denote significant difference from control and KT5832, respectively.

**Figure 3:**
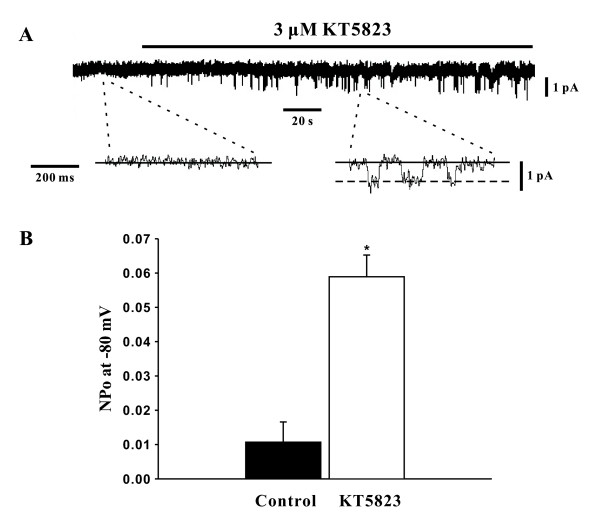
**PKG inhibition by KT5823 augments single channel SOC activity**. Using cell attached patch clamp electrophysiology, single channel SOC activity in PASMCs was assessed (holding potential, -80 mV) in the absence and presence of 3 μM KT5823. Representative traces and summary data can be found in **A **&**B**, respectively. Data are means ± SE, n = 6. * denotes significant difference from control.

To determine whether PKG can modulate SOCs, this study first employed whole cell patch clamp electrophysiology to monitor SOC currents in the absence of presence of KT5823. The representative trace in Figure 2A illustrates that the bath application of KT5823 (3 μM) elevated SOC current and that SOC inhibitors SKF-96365 (10 μM) and Ni^2+ ^(0.1 mM) [13,16] reversed and attenuated this effect respectively. As shown in Figure 2B, the mean inward current (at -80 mV) increased from -21.6 ± 1.4 pA to -138.8 ± 12.0 pA (n = 6, P < 0.01) in the presence of KT5823 and this effect was largely eliminated by SKF-96365 (-23.5 ± 2.1 pA, n = 6, P < 0.01) or Ni^2+ ^(-82.0 ± 10.1 pA, n = 6, P < 0.01). With whole cell measurements suggesting that PKG likely inhibits SOCs in PASMCs, the cell-attached configuration was subsequently employed to assess single channel SOC activity prior to and following PKG inhibition. Representative traces in Figure 3A show on two different time scales that the bath application of KT5823 (3 μM) increases SOC activity. Summary data in Figure 3B highlights that KT5823 elevated the mean open probability (NP_o_) from 0.0107 ± 0.0059 to 0.0589 ± 0.0063 (n = 6, P < 0.01).

### Activation of SOCs by a PKA inhibitor

**Figure 4:**
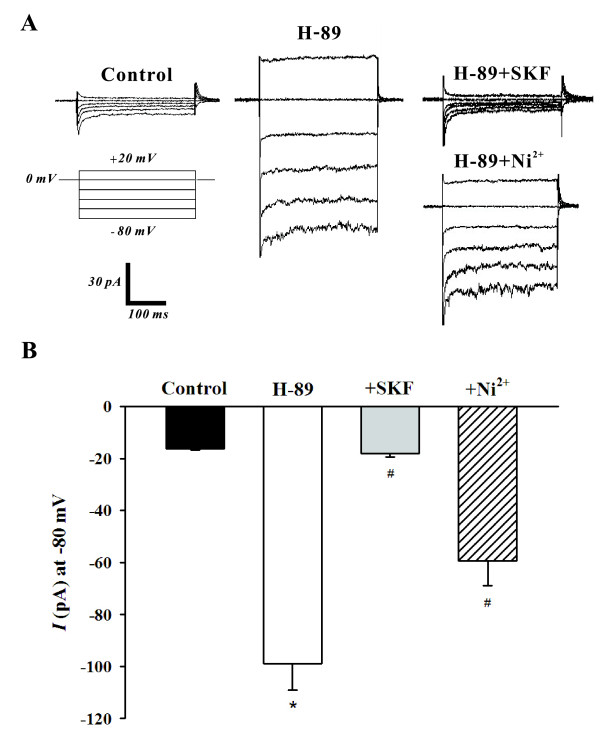
**PKA inhibition by H-89 augments SOC whole cell currents**. Using whole cell patch clamp electrophysiology, PASMCs were voltage clamped and then periodically exposed to a step protocol (-80 mV to +20 mV, 20 mV increments, 300 ms duration) in the absence and presence of 10 μM H-89 ± 10 μM SKF-96365 (SKF) or 0.1 mM Ni^2+^. A representative trace and summary data can be found in **A **&**B**, respectively. Data are means ± SE, n = 6. * and # denote significant difference from control and H-89, respectively.

**Figure 5:**
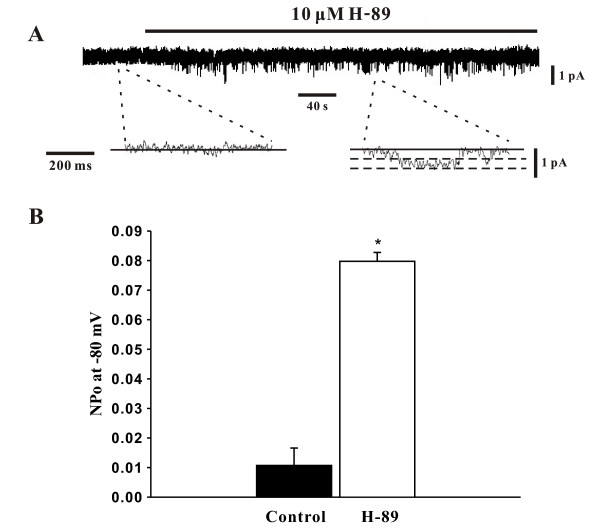
**PKA inhibition by H-89 augments single channel SOC activity**. Using cell attached patch clamp electrophysiology, single channel SOC activity in PASMCs was assessed (holding potential, -80 mV) in the absence and presence of 10 μM H-89. Representative traces and summary data can be found in **A **&**B**, respectively. Data are means ± SE, n = 6. * denotes significant difference from control.

To ascertain the role of PKA in the modulation of SOCs, this study again employed whole cell patch clamp electrophysiology to monitor SOC currents in the absence and presence of H-89. The representative trace in Figure 4A shows that the addition of H-89 (10 μM) significantly increased the SOC current and that the bath application of SKF-96365 (10 μM) and Ni^2+ ^(0.1 mM) abolished and attenuated this elevation respectively. Mean inward current (at -80 mV) plotted in Figure 4B further emphasized that the H-89 induced increase in SOC activity (-16.2 ± 0.5 pA to -98.8 ± 9.1 pA, n = 6, P < 0.01) was indeed effectively blocked by SKF-96365 (-17.5 ± 1.6 pA, n = 6, P < 0.01) or Ni^2+ ^(-59.4 ± 9.5 pA, n = 6, P < 0.01). With whole cell measurements indicating that PKA likely inhibits SOCs in PASMCs, the cell-attached configuration was once again employed to monitor single channel SOC activity. The representative trace in Figure 5A nicely illustrates, at two different time scales, that the bath application of H-89 (10 μM) elevates SOC activity. Mean NP_o _was plotted in Figure 5B and reinforces that single channel SOC activity rose from 0.0106 ± 0.0056 to 0.0798 ± 0.0028 (n = 6, P < 0.01) in the presence of H-89.

### Activation of SOCs by a PKC activator and IP_3_

**Figure 6:**
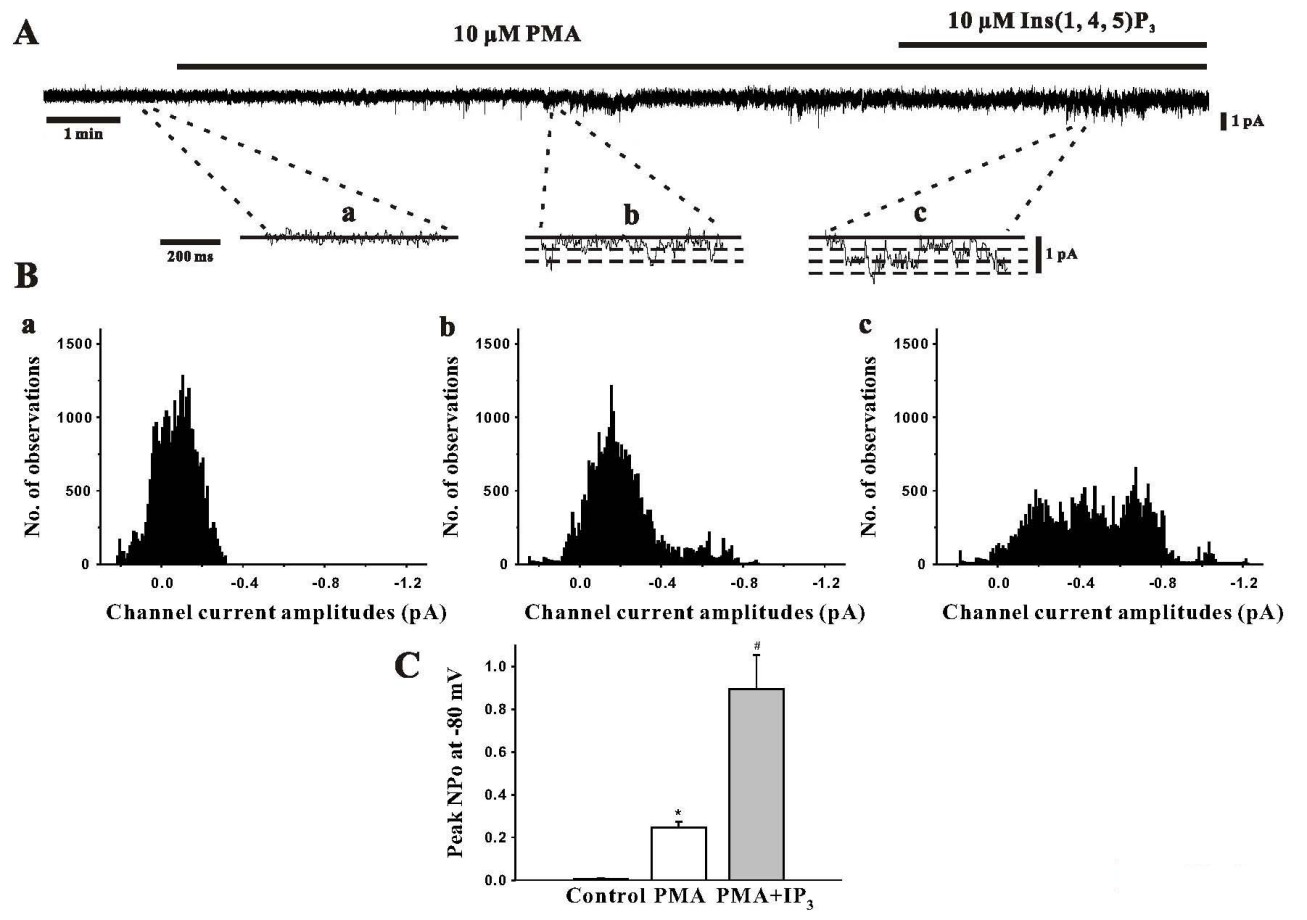
**Effects of PKC activation and Ins(1,4,5)P_3 _on single channel SOC activity**. Using inside out patch clamp electrophysiology, single channel SOC activity in PASMCs was assessed (holding potential, -80 mV) in the absence and presence of 10 μM PMA ± 10 μM Ins(1,4,5)P_3_. Representative traces and amplitude histograms of single channel currents (a, control; b, PMA; c, PMA + **Ins(1,4,5)P**_**3 **_can be found in **A **&**B**, respectively. Mean NP_o _data was plotted in **C**. Data are means ± SE, n = 6. * and # denote significant difference from control and PMA, respectively.

Finally, to study the involvement of PKC and possibly Ins(1,4,5)P_3 _in SOC modulation, the inside-out configuration was utilized to monitor these channels in the absence and presence of PMA (a PKC activator) and Ins(1,4,5)P_3_. As the representative traces in Figure 6A show, bath application of PMA (10 μM) induced a modest increase in SOC activity that was further augmented by the addition of Ins(1,4,5)P_3_. The amplitude and NP_o _histograms in Figure 6B and 6C statistical confirm this. Of particular note was the increase in NP_o _from 0.0056 ± 0.0023 to 0.2475 ± 0.0261 (P < 0.05) and to 0.8949 ± 0.1573 (n = 6, P < 0.05) as paired experiments advanced from rest, to PMA addition and finally to the dual application of PMA and Ins(1,4,5)P_3_. Cumulatively, these experiments support an important role for PKC and IP_3 _in the activation of SOCs in PASMCs.

## Discussion

**Figure 7:**
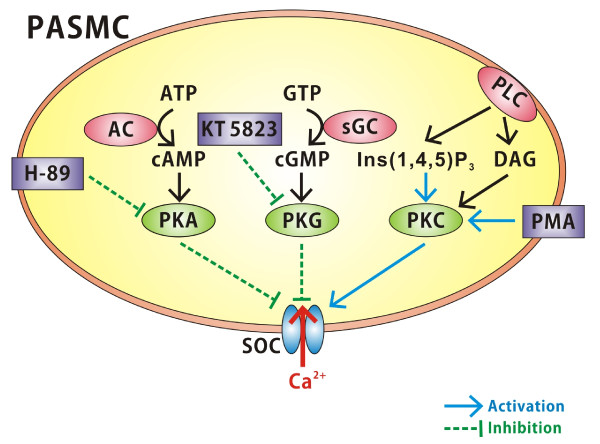
**Diagram highlighting protein kinase modulation of SOCs in rat PASMCs**. Note that protein kinase G (PKG) and protein kinase A (PKA) inhibit SOC currents whereas protein kinase C (PKC) appears to activate SOC currents. PKC activation of SOC currents is augmented by phospholipase C (PLC) activation and the production of **Ins(1,4,5)P**_**3**_. AC, adenylate cyclase; sGC, soluble guanylate cyclase.

This study is the first to use patch clamp electrophysiology to investigate the role of protein kinases in the modulation of SOCs in rat PASMCs. To briefly summarize, this study observed that PKG and PKA elicited an inhibitory effect on SOC channels when measured at the whole cell and single channel level. Conversely, PKC appears to activate these channels and this augmentation was enhanced by the addition of Ins(1,4,5)P_3 _(Figure 7). The findings show that SOCs in PASMCs are diversely targeted by protein kinases. Such regulation likely plays an important role in setting pulmonary arterial tone under normal and pathophysiological conditions.

### Type and character of SOCs in pulmonary artery myocytes

There are three types of SOCs described in vascular smooth muscles. *I*_SOC1 _is the conductance (γ = 2-3 pS) described in rabbit portal vein myocytes. *I*_SOC2 _is the conductance (γ = 3 pS) described in aortic smooth muscle. *I*_SOC3 _is the conductance described in mouse anococcygeus muscle due to its estimated conductance of less than 1 pS [4]. Single channel currents induced by CPA have also been recorded in cell-attached patches from cultured human PASMCs, which had a slope conductance of about 5 pS [17]. From the differences in the biophysical properties of SOCs recorded in smooth muscles, it is evident that there are different types of SOCs which probably reflect different molecular identities and possibly physiological functions [4].

SOCs play an important role in controlling Ca^2+ ^influx, arterial tone development and smooth muscle cell growth in the pulmonary vasculature [6,7]. While it is commonly agreed that these channels are activated by SR store depletion, their electrical properties do show some degree of variability. For example, the *I-V *relationship of SOC currents can be both linear in nature or outward rectifying [9]. Their relative permeability for monovalent and divalent cations has also been difficult to precisely define but appears to shift among published studies [17]. In this study, we report that CPA-evoked currents in rat PASMCs display, in essence, a linear *I-V *relationship at negative potentials and a limited degree of outward rectification at positive potentials. Although relative cation permeability was not directly ascertained, the composition of our recording solutions dictates that at negative potentials, Na^+^/Ca^2+ ^influx should dominate the whole cell current where as Cs^+ ^efflux will dominate at positive potentials. As such, the SOC channels noted in this investigation share some of the same biophysical characteristics as those previous isolated from rat and human pulmonary arteries [17,18].

### Activation of SOCs in pulmonary artery myocytes is regulated by PKG and PKA

The guanylate cyclase-cyclic GMP-protein kinase G signaling pathway plays a pivotal role in several physiological processes including vascular tone development and cell cycle progression. Broadly speaking, studies have shown that activation of this NO-dependent signaling pathway characteristically attenuates SOC activity in smooth muscle [19-21]. This is exemplified by the inhibition of SOCs not only in A7R5 cells [22] but in native smooth muscle cells derived from the mouse anococcygeus and the rat systemic circulation [4,23,24]. In HEK-293 cells expressing recombinant canonical transient receptor potential isoform 3 (TRPC3), protein kinase G was reported to phosphorylate and inhibit Ca^2+ ^influx through TRPC3 channels, the latter apparently functioning in a store-operated mode [20,25]. On the other hand, cGMP did not affect the inactivation of Ca^2+ ^release-activated Ca^2+ ^current (I_CRAC_) in RBL-1 cells [26], nor did membrane-permeable analogs of cGMP alter store-operated Ca^2+ ^entry (SOCE) in *Xenopus *oocytes [27], pancreatic acinar cells [28], or T lymphocytes [29]. Regulation of SOCE by cGMP is likely therefore to be cell type specific [25]. In a similar manner, it is thought that the adenylate cyclase-cyclic AMP-PKA signaling pathway relaxes vascular smooth muscle in part due to the inhibition of SOCs [30]. Indeed, in portal vein myocytes, β-adrenoceptor stimulation attenuates SOC activity whereas PKA inhibitors (H-89 and KT5720) elicit the reciprocal effect [31,32]. With regard to PKA-mediated inhibition of SOCs in portal vein myocytes there is some similarity to data obtained in *Xenopus *oocytes [27,31]. Petersen and Berridge [27] demonstrated that low concentrations of dibutyryl cAMP (1-10 μM) inhibited Ca^2+ ^influx but higher concentrations (1-10 mM) potentiated SOCE. It should be noted that in corneal epithelial cells PKA has also been shown to inhibit an epidermal growth factor-evoked Ca^2+ ^influx pathway, attributed to opening of SOCs [33]. In this study, we extended the idea that both the NO-cGMP-protein kinase G and cAMP-protein kinase A signaling pathways effectively regulate SOCs by demonstrating that PKG and PKA inhibition (KT5823 and H-89, respectively) augment single channel activity in PASMCs. From a physiological perspective, this inhibition would likely raise intracellular calcium ([Ca^2+^]_i_), an event intimately tied to both contraction and proliferation in PASMCs. Such alterations could in turn contribute to the constriction and medial hypertrophy that commonly underlies pulmonary arterial hypertension (PAH).

### Activation of SOCs in pulmonary artery myocytes is regulated by PKC and IP_3_

Past studies have strongly linked PKC activation to the augmentation of SOC activity in vascular smooth muscle [32]. Particularly noteworthy has been the ability of phorbol esters and 1-oleoyl-sn-glycerol (diacylglycerol analogue) to activate and PKC inhibitors to attenuate SOCs [4,8,34]. Interestingly, this PKC-induced activation of vascular SOCs appears to be augmented by the production of IP_3 _and/or the depletion of PIP_2 _[2,35]. Such observations suggest some degree of cooperation among the various signaling events activated by vasoconstrictors via G-protein coupled receptors. In canine pulmonary vein smooth muscle cells, activation or inhibition of PKC was found to have no effect on SOCE [36]; thus, there appears to be considerable diversity in the role of PKC plays in regulating this Ca^2+ ^entry pathway in different cells [34]. Perhaps SOCE pathways differ in different blood vessels. Indeed, smooth muscle cells from mesenteric and coronary arteries have store depletion activated cation channels with distinct properties, although both are activated by PKC [8]. A wide range of properties have been reported for SOCs in different smooth muscle preparations, suggesting that multiple cation channels can be opened by store depletion [37,38]. In this study of PASMCs, we observed a similar phenomenon whereby the PMA-induced increase in SOC activity was further enhanced by the application of IP_3_. Generally speaking, in physiological states this activation would be more important than PKG and PKA inhibition to induce both the contraction and proliferation in PASMCs, which promotes the development of severe PAH.

## Limitations

It is generally agreed that SOCs are relatively difficult to isolate from other conductance channels in the plasma membrane because of a deficiency of highly selective pharmacological agents and the lack of selectively characteristic electrophysiological properties. A previous report [4] demonstrated that cell-attached recording may be an improved method for studying SOCs in smooth muscle, and benefits from the advantage of not disturbing the intracellular milieu. In this study, we initially showed that PKG and PKA inhibition and PKC activation enhance the SOC currents in freshly dispersed rat PASMCs. Nevertheless, over the time course of typical experiments, we cannot exclude the possibility that an induced current of such amplitude is due to a change in 'leak' which might occur, for example, due to myocyte contraction. Accordingly, to further confirm our findings, cell-attached and inside-out configurations were used to measure single channel SOC activity with and without PKG and PKA inhibitors (KT5823 and H-89) and PKC activator (PMA) and IP_3_. The data obtained from single channel SOC activity was consistent with those of whole cell SOC currents. However, we still need to interpret our findings cautiously because the supposed opposite effects on SOC channels with activators of PKG and PKA and/or inhibitors of PKC in rat PASMCs remain unresolved. Also, it is not yet clear what upstream and/or downstream signaling molecules are involved in these protein kinase pathways.

## Conclusions

In summary, this study presents evidence that protein kinases play an important role in regulating SOCs in smooth muscles derived from the pulmonary artery. While McElroy et al. [34] previously used Ca^2+^-imaging technique to show that protein kinases can regulate SOCs in rat PASMCs, this investigation appears to be the first to use patch-clamp electrophysiology to measure similar SOC regulation at the whole cell and single channel level. Given that enhanced SOC activity has been linked to the development of pulmonary arteriopathies, we proposed that protein kinase modulation may provide a means of attenuating the progression of these debilitating disorders.

## Abbreviations

[Ca^2+^]_i_: intracellular calcium; CCE: capacitative Ca^2+ ^entry; CIF: calcium influx factor; CPA: cyclopiazonic acid; I_CRAC_: Ca^2+ ^release-activated Ca^2+ ^current; iPLA_2_: Ca^2+^-independent phospholipase A_2_; Ins(1,4,5)P_3_: inositol 1,4,5-triphosphate; PASMC: pulmonary artery smooth muscle cell; PKA: protein kinase A; PKC: protein kinase C; PKG: protein kinase G; PMA: phorbol 12-myristate 13-acetate; SERCA: sarco-endoplasmic reticulum Ca^2+^-ATPase pump; SOC: store-operated channel; SOCE: store-operated Ca^2+ ^entry; TRPC3: canonical transient receptor potential isoform 3

## Competing interests

The authors declare that they have no competing interests.

## Authors' contributions

ISC performed the experiments and drafted the manuscript. ZKD and IJC provided the ideas and participated in the design and coordination of this study, and helped to draft the manuscript. DGW and BNW designed and directed the experiments, interpreted the data and polished the paper to meet the scientific content. All authors read and approved the final manuscript.

## References

[B1] Salido GM Sago SO Rosado JA TRPC channels and store-operated Ca^2+ ^entry Biochim Biophys Acta 2008 1793 223 230 19061922 10.1016/j.bbamcr.2008.11.001

[B2] Liu M Albert AP Large WA Facilitatory effect of Ins(1,4,5)P_3 _on store-operated Ca^2+^-permeable cation channels in rabbit portal vein myocytes J Physiol 2005 566 161 171 10.1113/jphysiol.2005.088260 15860523 PMC1464740

[B3] Putney JW Jr A model for receptor-regulated calcium entry Cell Calcium 1986 7 1 12 10.1016/0143-4160(86)90026-6 2420465

[B4] Albert AP Large WA Store-operated Ca^2+^-permeable non-selective cation channels in smooth muscle cells Cell Calcium 2003 33 345 356 10.1016/S0143-4160(03)00048-4 12765681

[B5] Smani T Zakharov SI Csutora P Leno E Trepakova ES Bolotina VM A novel mechanism for the store-operated calcium influx pathway Nat Cell Biol 2004 2 113 120 10.1038/ncb1089 14730314

[B6] Landsberg JW Yuan JX Calcium and TRP channels in pulmonary vascular smooth muscle cell proliferation News Physiol Sci 2004 19 44 50 15016901 10.1152/nips.01457.2003

[B7] Lu W Ran P Zhang D Peng G Li B Zhong N Wang J Sildenafil inhibits chronically hypoxic upregulation of canonical transient receptor potential expression in rat pulmonary arterial smooth muscle Am J Physiol Cell Physiol 2010 298 C114 C123 10.1152/ajpcell.00629.2008 19889962 PMC2806156

[B8] Saleh SN Albert AP Peppiatt CM Large WA Diverse properties of store-operated TRPC channels activated by protein kinase C in vascular myocytes J Physiol 2008 586 2463 2476 10.1113/jphysiol.2008.152157 18356201 PMC2408673

[B9] Ng LC Gurney AM Store-operated channels mediate Ca^2+ ^influx and contraction in rat pulmonary artery Circ Res 2001 89 923 929 10.1161/hh2201.100315 11701620

[B10] Hamill OP Marty A Neher E Sakmann B Sigworth FJ Improved patch-clamp techniques for high-resolution current recording from cells and cell-free membrane patches Pflugers Arch 1981 391 85 100 10.1007/BF00656997 6270629

[B11] Luykenaar KD El-Rahman RA Walsh MP Welsh DG Rho-kinase-mediated suppression of K_DR _current in cerebral arteries requires an intact actin cytoskeleton Am J Physiol Heart Circ Physiol 2009 296 H917 H926 10.1152/ajpheart.01206.2008 19218502 PMC2670701

[B12] Wu BN Luykenaar KD Brayden JE Giles WR Corteling RL Wiehler WB Welsh DG Hyposmotic challenge inhibits inward rectifying K^+ ^channels in cerebral arterial smooth muscle cells Am J Physiol Heart Circ Physiol 2007 292 H1085 H1094 10.1152/ajpheart.00926.2006 17056667

[B13] Yu Y Sweeney M Zhang S Platoshyn O Landsberg J Rothman A Yuan JX PDGF stimulates pulmonary vascular smooth muscle cell proliferation by upregulating TRPC6 expression Am J Physiol Cell Physiol 2003 284 C316 C330 12529250 10.1152/ajpcell.00125.2002

[B14] Ng LC McCormack MD Airey JA Singer CA Keller PS Shen XM Hume JR TRPC1 and STIM1 mediate capacitative Ca^2+ ^entry in mouse pulmonary arterial smooth muscle cells J Physiol 2009 587 2429 2442 10.1113/jphysiol.2009.172254 19332490 PMC2714011

[B15] Smith PD Brett SE Luykenaar KD Sandow SL Marrelli SP Vigmond EJ Welsh DG K_IR _channels function as electrical amplifiers in rat vascular smooth muscle J Physiol 2008 586 1147 1160 10.1113/jphysiol.2007.145474 18063660 PMC2375635

[B16] Wilson SM Mason HS Smith GD Nicholson N Johnston L Janiak R Hume JR Comparative capacitative calcium entry mechanisms in canine pulmonary and renal arterial smooth muscle cells J Physiol 2002 543 917 931 10.1113/jphysiol.2002.021998 12231648 PMC2290529

[B17] Golovina VA Platoshyn O Bailey CL Wang J Limsuwan A Sweeney M Rubin LJ Yuan JX Upregulated TRP and enhanced capacitative Ca^2+ ^entry in human pulmonary artery myocytes during proliferation Am J Physiol Heart Circ Physiol 2001 280 H746 H755 11158974 10.1152/ajpheart.2001.280.2.H746

[B18] Guibert C Marthan R Savineau JP 5-HT induces an arachidonic acid-sensitive calcium influx in rat small intrapulmonary artery Am J Physiol Lung Cell Mol Physiol 2004 286 L1228 L1236 10.1152/ajplung.00265.2003 14751848

[B19] Moneer Z Dyer JL Taylor CW Nitric oxide co-ordinates the activities of the capacitative and non-capacitative Ca^2+^-entry pathways regulated by vasopressin Biochem J 2003 370 439 448 10.1042/BJ20021104 12459038 PMC1223200

[B20] Kwan HY Huang Y Yao X Regulation of canonical transient receptor potential isoform 3 (TRPC3) channel by protein kinase G Proc Natl Acad Sci USA 2004 101 2625 2630 10.1073/pnas.0304471101 14983059 PMC357000

[B21] Yao X TRPC, cGMP-dependent protein kinases and cytosolic Ca^2+^ Handbook Exp Pharmacol 2007 179 527 540 full_text 10.1007/978-3-540-34891-7_31 17217077

[B22] Moneer Z Taylor CW Reciprocal regulation of capacitative and non-capacitative Ca^2+ ^entry in A7r5 vascular smooth muscle cells: only the letter operates receptor activation Biochem J 2002 362 13 21 10.1042/0264-6021:3620013 11829735 PMC1222355

[B23] Wayman CP McFadzean I Gibson A Tucker JF Two distinct membrane currents activated by cyclopiazonic acid-induced calcium store depletion in single smooth muscle cells of the mouse anococcygeus Br J Pharmacol 1996 117 566 572 8821550 10.1111/j.1476-5381.1996.tb15228.x PMC1909300

[B24] Inoue R Jian Z Kawarabayashi Y Mechanosensitive TRP channels in cardiovascular pathophysiology Pharmacol Ther 2009 123 371 385 10.1016/j.pharmthera.2009.05.009 19501617

[B25] Parekh AB Putney JW Jr Store-operated calcium channels Physiol Rev 2005 85 757 810 10.1152/physrev.00057.2003 15788710

[B26] Parekh AB Penner R Depletion-activated calcium current is inhibited by protein kinase in RBL-2H3 cells Proc Natl Acad Sci USA 1995 92 7907 7911 10.1073/pnas.92.17.7907 7644512 PMC41255

[B27] Petersen CC Berridge MJ G-protein regulation of capacitative calcium entry may be mediated by protein kinases A and C in Xenopus oocytes Biochem J 1995 307 663 668 7741694 10.1042/bj3070663 PMC1136702

[B28] Gilon P Obie JF Bian X Bird GS Putney JW Jr Role of cyclic GMP in the control of capacitative Ca^2+ ^entry in rat pancreatic acinar cells Biochem J 1995 311 649 656 7487909 10.1042/bj3110649 PMC1136049

[B29] Bian X Bird GS Putney JW Jr cGMP is not required for capacitative Ca^2+ ^entry in Jurkat T-lymphocytes Cell Calcium 1996 19 351 354 10.1016/S0143-4160(96)90075-5 8983855

[B30] Smani T Domínguez-Rodríguez A Hmadcha A Calderón-Sánchez E Horrillo-Ledesma A Ordóñez A Role of Ca^2+^-independent phospholipase A_2 _and store-operated pathway in urocortin-induced vasodilatation of rat coronary artery Circ Res 2007 101 1194 1203 10.1161/CIRCRESAHA.107.159053 17885217

[B31] Liu M Large WA Albert AP Stimulation of β-adrenoceptors inhibits store-operated channel currents via a cAMP-dependent protein kinase mechanism in rabbit portal vein myocytes J Physiol 2005 562 395 406 10.1113/jphysiol.2004.077602 15528235 PMC1665505

[B32] Albert AP Saleh SN Peppiatt CM Large WA Multiple activation mechanisms of store-operated TRPC channels in smooth muscle cells J Physiol 2007 583 25 36 10.1113/jphysiol.2007.137802 17615095 PMC2277241

[B33] Yang H Sun X Wang Z Ning G Zhang F Kong J Lu L Reinach PS EGF stimulates growth by enhancing capacitative calcium entry in corneal epithelial cell J Memb Biol 2003 194 47 58 10.1007/s00232-003-2025-9 14502442

[B34] McElroy SP Drummond RM Gurney AM Regulation of store-operated Ca^2+ ^entry in pulmonary artery smooth muscle cells Cell Calcium 2009 46 99 106 10.1016/j.ceca.2009.05.006 19573912

[B35] Saleh SN Albert AP Large WA Activation of native TRPC1/C5/C6 channels by endothelin-1 is mediated by both PIP(3) and PIP(2) in rabbit coronary artery myocytes J Physiol 2009 587 5361 5375 10.1113/jphysiol.2009.180331 19770190 PMC2788089

[B36] Shimizu S Ding X Murray PA Intravenous anesthetics inhibit capacitative calcium entry in pulmonary venous smooth muscle cells Anesthesiology 2006 104 791 797 10.1097/00000542-200604000-00025 16571976

[B37] Beech DJ Muraki K Flemming R Non-selective cationic channels of smooth muscle and the mammalian homologues of Drosophila TRP J Physiol 2004 559 685 706 15272031 10.1113/jphysiol.2004.068734 PMC1665181

[B38] Leung FP Yung LM Yao X Laher I Huang Y Store-operated calcium entry in vascular smooth muscle Br J Pharmacol 1996 153 846 857 10.1038/sj.bjp.0707455 PMC2267267 17876304

